# Thermophilic *Moorella thermoacetica* as a platform microorganism for C1 gas utilization: physiology, engineering, and applications

**DOI:** 10.1186/s40643-023-00682-z

**Published:** 2023-09-15

**Authors:** Dechen Jia, Wangshuying Deng, Peng Hu, Weihong Jiang, Yang Gu

**Affiliations:** 1 CAS-Key Laboratory of Synthetic Biology, CAS Center for Excellence in Molecular Plant Sciences, Institute of Plant Physiology and Ecology, Chinese Academy of Sciences, Shanghai, 200032 China; 2 University of Chinese Academy of Sciences, Beijing, 100049 China; 3 Shanghai GTLB Biotech Co., Ltd, 1688 North Guoquan Road, Shanghai, 200438 China

**Keywords:** *Moorella thermoacetica*, C1 gases, Physiology and metabolism, Genetic tools, Strain improvements

## Abstract

In the context of the rapid development of low-carbon economy, there has been increasing interest in utilizing naturally abundant and cost-effective one-carbon (C1) substrates for sustainable production of chemicals and fuels. *Moorella thermoacetica*, a model acetogenic bacterium, has attracted significant attention due to its ability to utilize carbon dioxide (CO_2_) and carbon monoxide (CO) via the Wood–Ljungdahl (WL) pathway, thereby showing great potential for the utilization of C1 gases. However, natural strains of *M. thermoacetica* are not yet fully suitable for industrial applications due to their limitations in carbon assimilation and conversion efficiency as well as limited product range. Over the past decade, progresses have been made in the development of genetic tools for *M. thermoacetica*, accelerating the understanding and modification of this acetogen. Here, we summarize the physiological and metabolic characteristics of *M. thermoacetica* and review the recent advances in engineering this bacterium. Finally, we propose the future directions for exploring the real potential of *M. thermoacetica* in industrial applications.

## Introduction

C1 gases such as CO_2_ and CO are abundant and cost-effective carbon resources that can be derived from various sources, including industrial off-gases (e.g., steel manufacture, and oil refining and coal chemical industries) or through gasification of forestry and agricultural wastes (De Tissera et al. 2019). In addition, CO_2_ is a major greenhouse gas that contributes to global warming (Balcombe et al. 2018). Consequently, the capture and utilization of these C1 gases have attracted great attention due to the need to reduce greenhouse gas emission and achieve sustainable production of chemicals and fuels with minimal and even negative carbon footprint.

In comparison with chemical catalysis, microbial conversion has specific advantages in the utilization of C1 gases, particularly in the synthesis of medium and long-carbon chain products (Bae et al. 2022; Liu et al. 2020a). For example, acetogenic bacteria have ability to capture C1 gases and convert them into multiple products using different energy forms (Liao et al. 2016). Furthermore, several acetogenic bacteria can use both C1 gases (CO and CO_2_) and liquid C1 sources, such as formate, showcasing a wide substrate range (Jia et al. 2021; Neuendorf et al. 2021).

*Moorella thermoacetica,* initially known as *Clostridium thermoaceticum* due to its morphological and physiological similarities to *Clostridium* species (Fontaine et al. 1942), is one of the earliest isolated acetogens (Drake et al. 2008). However, in the late 1990s, it was officially renamed *M. thermoacetica* during the reclassification of the genus *Clostridium* (Collins et al. 1994). Over time, a series of *M. thermoacetica* strains have been identified (Table 1), in which some have received extensive concern (Redl et al. 2020; Sakai et al. 2005; Wang and Wang 1984). As a thermophilic bacterium, *M. thermoacetica* normally grows within 45‒65 °C, with an optimum temperature of 55–60 °C. The thermophilic characteristic of *M. thermoacetica* reduces the risk of bacterial contamination during its fermentation (Kato et al. 2021). Despite showcasing the potential in the utilization of C1 gases, natural *M. thermoacetica* strains still have large promotion space in carbon conversion efficiency and product yield. Consequently, much effort has been taken in the design and construction of artificial *M. thermoacetica* strains with the advent of suitable genetic tools for this bacterium in the past decade.

**Table 1:** Physiology and traits of major *M. thermoacetica* strains

Organisms	Genome Size (Mbp)	Carbon sources	Optimal temperature (℃)	Products^*a*^	References
HUC22-1	‒	H_2_/CO_2_, fructose	55	840 mM acetate, 15.4 mM ethanol	(Sakai et al. 2005, 2004)
ATCC 31490	Contig (2.61680)	H_2_/CO_2_, fructose, glucose	60	48.3 mM	(Redl et al. 2020; Schwartz Robert and Keller Jr Frederick 1980)
ATCC 33924	Contig (2.91484)	Xylose, CO, CO_2_	55	222 mM acetate	(Redl et al. 2020; Savage et al. 1987)
ATCC 39073	Complete (2.62878)	Xylose, fructose, glucose, H_2_/CO_2_, CO, pyruvate, formate, vanillate	55	933 mM acetate, 10 mM acetone	(Fröstl et al. 1996; Kato et al. 2021; Poehlein et al. 2015; Redl et al. 2020; Rosenbaum et al. 2021; Schaible 1997; Wang & Wang 1984)
ATCC 39073-HH	Complete (2.64566)	Sucrose, xylose, fructose, glucose, H_2_/CO_2_, methanol, pyruvate	55	−	(Redl et al. 2020)
ATCC 39289	−	Xylose, glucose, pyruvate, formate	55	750 mM acetate	(Keller Jr Frederick et al. 1983; Reed William 1984)
ATCC 49707	Contig (2.61685)	Glucose, fructose, xylose, H_2_/CO_2_	55	401.7 mM acetate	(Andreesen et al. 1973; Brumm Phillip and Datta 1985; Redl et al. 2020; Schaible 1997)
DSM 103132	Complete (2.97608)	Sucrose, arabinose, formate, fructose, glucose, H_2_/CO_2_, methanol, pyruvate,	60	−	(Redl et al. 2020)
DSM 103284	Complete (2.56038)	Xylose, fructose, glucose, H_2_/CO_2_, methanol, pyruvate, rhamnose, xylose	60	−	(Redl et al. 2020)
DSM 11768	Contig (2.85144)	Fructose	60	−	(Redl et al. 2020)
DSM 12797	Contig (2.74601)	H_2_/CO_2_, CO/CO_2_, lactate, formate, cellobiose, fructose, glucose	60	18.4 mM acetate, 13.7 mM succinate, 1.8 mM lactate, 8.6 mM ethanol, 2.4 mM formate	(Gößner et al. 1999; Redl et al. 2020)
DSM 12993	Contig (2.64895)	Fructose	60	−	(Redl et al. 2020)
DSM 2955	Complete (2.62335)	Xylose, fructose, glucose, H_2_/CO_2_, methanol, pyruvate, CO	60	−	(Bengelsdorf et al. 2015; Redl et al. 2020)
Y72	Scaffold (2.60381)	Xylose	60	−	(Tsukahara et al. 2014)

^−^no available data

^*a*^The highest product levels reported for corresponding strains

In this review, we summarize the current knowledge regarding the physiological and metabolic characteristics of *M. thermoacetica*. We also discuss the recent progresses in metabolic design, engineering, and fermentation optimization of this acetogen. While genetic tools are currently available for *M. thermoacetica*, we propose the direction of further optimizing the toolbox for efficient strain modification and improvement. Furthermore, we highlight the future challenges that need to be addressed to fully explore the real potential of *M. thermoacetica* in C1 gas utilization.

## Broad carbon substrate range of *M. thermoacetica*

*Moorella* *thermoacetica* exhibits versatility in its substrate spectrum, ranging from CO_2_/CO to various hexose and pentose sugars (Table 1). In gas fermentation, this bacterium converts two molecules of CO_2_ or CO into one molecule of acetyl-CoA through the Wood–Ljungdahl (WL) pathway (also referred to as the reductive acetyl-CoA pathway) and further transforms acetyl-CoA to multiple end products. Currently, the WL pathway is known as the shortest and least energy-consuming native carbon fixation pathway in organisms (Drake et al. 2008). Unlike CO_2_, CO can be utilized by acetogenic bacteria as both carbon and energy sources, and thus, the overall reactions for converting CO and CO_2_/H_2_ to end products (acetic acid and ethanol) via the WL pathway are different (Ukpong et al. 2012). The carbon yield in fermenting CO (as the sole carbon and energy sources) is lower than the case using CO_2_ and H_2_ as the carbon and energy source, respectively, because under the former condition a portion of CO has to be oxidized to CO_2_ to generate reducing force. Therefore, the complete utilization of CO by *M. thermoacetica* will largely depend on its reuse of the CO_2_ derived from CO, which requires the supplementation of extra energy, such as H_2_. For example, by adding H_2_ to CO, the product synthesis of *M. thermoacetica* from the same amount of carbon source was enhanced compared to the case of using CO only (Kato et al. 2021).

**Fig. 1:**
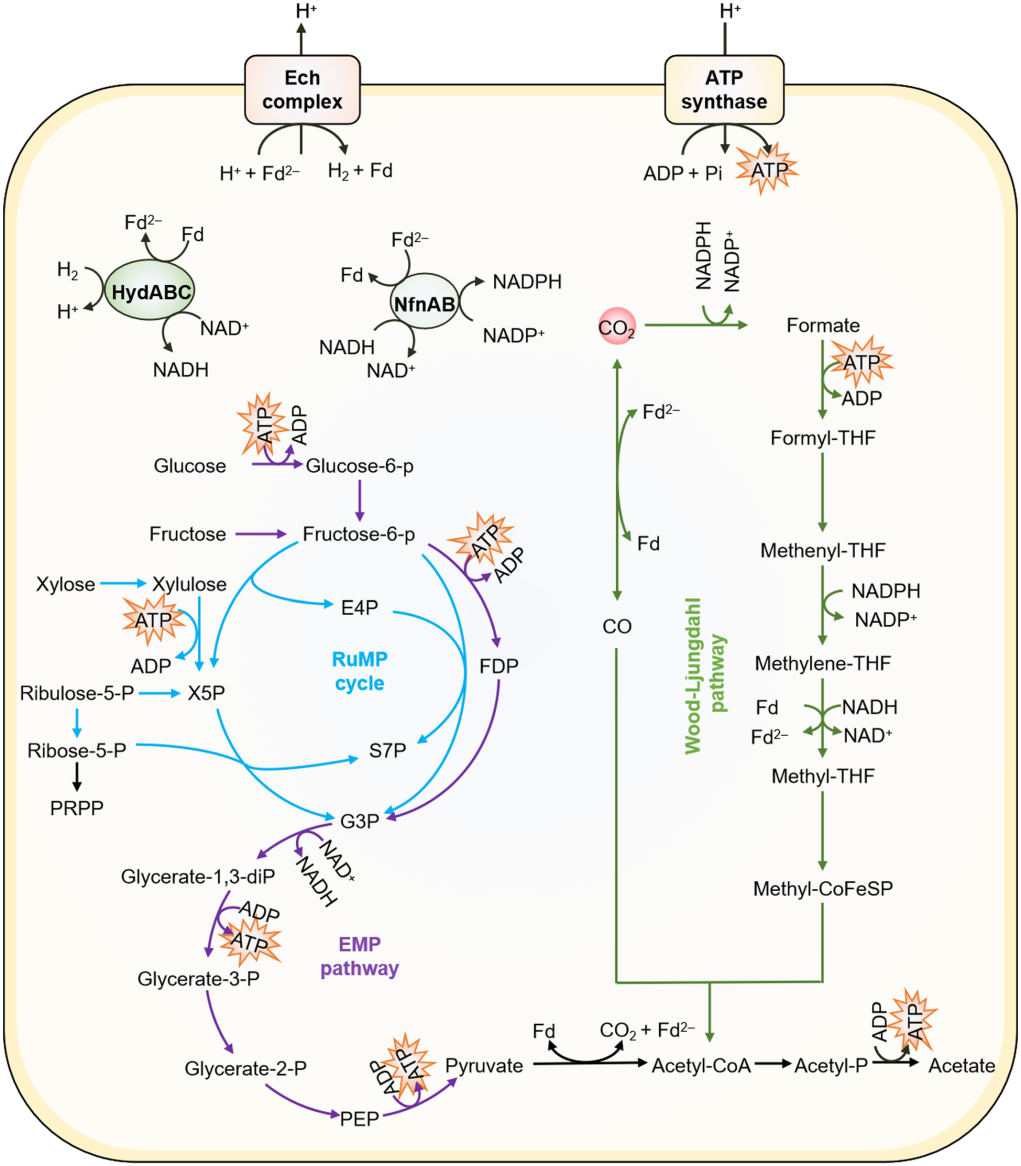
Model for carbon metabolism and energy conservation in *M. thermoacetica*. The RuMP cycle (blue), EMP pathway (purple) and Wood–Ljungdahl pathway (green) are necessary for growth on pentoses, hexoses, and C1 gases, respectively. Abbreviations of the different metabolites are as follows: THF, tetrahydrofolate; X5P, xylulose-5-p; E4P, erythrose-4-P; S7P, sedoheptulose 7-P; G3P, glyceraldehyde-3-P; FDP, fructose 1, 6-diphosphate; PEP, phosphoenolpyruvate; PRPP, 5-phosphoribosyl 1-pyrophosphate. Ech, energy-converting hydrogenase complex; HydABC, FeFe-hydrogenase; NfnAB, NADH-dependent reduced ferredoxin: NADP oxidoreductase

In addition to CO_2_/CO, *M. thermoacetica* can use multiple sugars (e.g., glucose, xylose, galactose, mannose, fructose, and arabinose) as the carbon sources (Andreesen et al. 1973; Ehsanipour et al. 2016; Fontaine et al. 1942). Importantly, with the assistance of the WL pathway, the CO_2_ released during glycolysis can be reassimilated by *M. thermoacetica*, thereby stoichimetrically converting one mole of glucose to three moles of acetate (Fig. 1). Such a high carbon yield surpasses that of typical heterotrophs and is also a common trait of acetogenic bacteria (Fast et al. 2015). Furthermore, all the candidate genes responsible for xylose and arabinose metabolism can be found in *M. thermoacetica* (Pierce et al. 2008), thus enabling this bacterium to utilize these two pentoses. As xylose and arabinose are the two major pentose sugars in lignocellulosic hydrolysates, *M. thermoacetica* has been employed in the use of these feedstocks for ethanol production (Rahayu et al. 2017, 2020).

Furthermore, *M. thermoacetica* have been found to metabolize many non-sugar organic compounds, such as formate, glyoxylate, glycolate, pyruvate, and lactate (Daniel and Drake 1993; Seifritz et al. 1999). Among them, formate and methanol are particularly attractive, because they can readily obtained from CO_2_/CO through chemical or whole-cell catalysis (Hwang et al. 2020; Jiang et al. 2021). It is known that formate is the reduced product from CO_2_ in the WL pathway (Fig. 1); thus, *M. thermoacetica* appears to be a potential platform microorganism for formate bioconversion, although further improvements in its tolerance and consumption to formate are needed. Regarding methanol, its assimilation in microorganisms typically employs two approaches: integration of methanol into the WL pathway via methanol methyltransferase, and oxidization of methanol to formaldehyde and formate, which are further assimilated through downstream pathways (Bae et al. 2022; Litty et al. 2022). The former approach has been identified in a representative acetogenic bacterium, *Acetobacterium woodii* (Litty et al. 2022; Wang et al. 2023). Whether these two approaches both function in the methanol metabolism in *M. thermoacetica* remains unclear.

## Product synthesis of *M. thermoacetica*

**Fig. 2:**
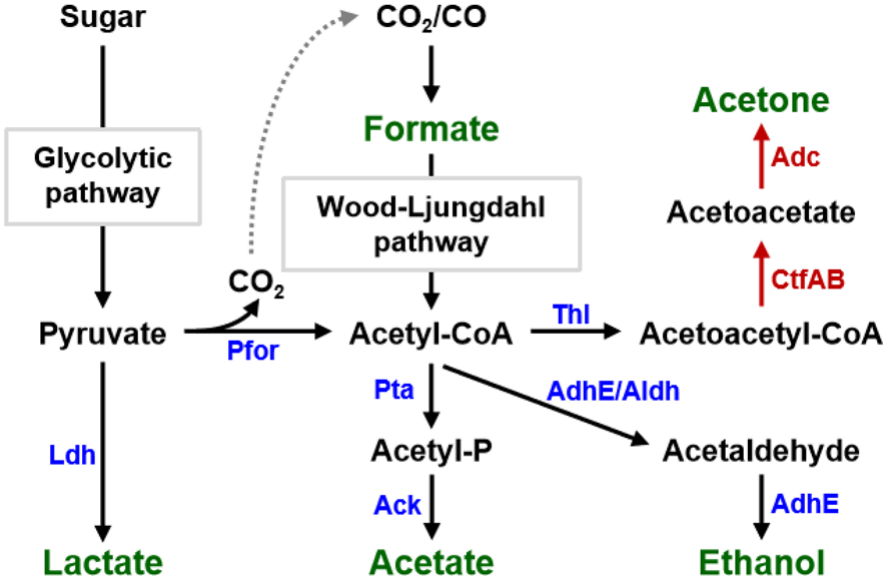
Products and related synthesis pathways in *M. thermoacetica.* All the reported products formed in *M. thermoacetica* are shown in green. The endogenous and heterologous enzymes responsible for the synthesis of these products are shown in blue and red, respectively. Abbreviations of the different enzymes are as follows: *Ack* acetate kinase, *AdhE* Acetaldehyde/alcohol dehydrogenase, *Aldh* Acetaldehyde dehydrogenase, *Adc* Acetoacetate decarboxylase, *CtfAB* Acetoacetyl-CoA transferase, *Ldh* Lactate dehydrogenase, *Pfor* Pyruvate ferredoxin oxidoreductase, *Pta* Phosphotransacetylase, *Thl* Thiolase

The native fermentation products of *M. thermoacetica* are limited (Fig. 2), with acetate being the predominant metabolite, typically accounting for over 90% of the total products (Wu et al. 2021). Some other minor products have also been observed in *M. thermoacetica* (Xue et al. 2014). The highest acetate production reported for *M. thermoacetica* was over 50 g/L during glucose fermentation via a fed-batch fermentation process (Parekh and Cheryan 1991). When grown on C1 gases, the acetate production level of this bacterium was also impressive. For instance, the *M. thermoacetica* ATCC 49707 strain could produce 31 g/L acetate with a productivity of 0.55 g/L/h by fermenting a mixture of CO and CO_2_ (Hu et al. 2013). Interestingly, neither class I nor II phosphotransacetylase (crucial enzymes converting acetyl-CoA to acetate in acetogens) have been found in *M. thermoacetica* based on its genome information (Pierce et al. 2008); instead, an atypical class III phosphotransacetylase has been identified (Breitkopf et al. 2016), indicating a unique mechanism for acetate synthesis in this bacterium. In addition, since acetate is an inexpensive chemical and can be easily utilized by many microorganisms, such as *Escherichia coli*, *Saccharomyces cerevisiae*, and *Corynebacterium glutamicum* (Chang et al. 2021; Chen et al. 2021; Huang et al. 2019, 2018; Lai et al. 2021; Liu et al. 2011; Merkel et al. 2022; Wei et al. 2015; Xu et al. 2021; Yang et al. 2019, 2020; Zhang et al. 2016), a two-step bioconversion approach for C1 gas utilization has been proposed, in which *M. thermoacetica* is responsible for producing acetate from CO_2_/CO and the generated acetate is subsequently converted into other value-added products by acetate-utilizing microorganisms (Huang et al. 2019, 2018; Lai et al. 2021; Liu et al. 2011; Yang et al. 2019, 2020).

*M. thermoacetica* is also capable of producing ethanol when it was grown on CO_2_ or CO. In acetogens, ethanol synthesis is normally dependent on two pathways: (i) conversion of acetyl-CoA to ethanol by aldehyde/alcohol dehydrogenases encoded by *adhE* genes; (ii) acetate transformation to acetaldehyde mediated by aldehyde oxidoreductase (Aor) followed by the conversion of acetaldehyde to ethanol (Liu et al. 2020b). It has been observed that the overexpression of the *aor* gene could effectively reduce acetate accumulation and improve ethanol production in acetogens (Jia et al. 2021). However, the deletion of the two *aor* genes in acetogenic *Clostridium autoethanogenum* led to different changes in ethanol production (Liew et al. 2017)*,* indicating the complexity of Aor's function in acetogens, which remains to be determined.

Besides acetic acid and ethanol, *M. thermoacetica* can produce a small amount of lactate, the monomer for the synthesis of polylactic acid (PLA) (Pierce et al. 2008). A bidirectional NAD^+^-dependent lactate dehydrogenase has been identified in *M. thermoacetica* with the optimum reaction temperature of 65 °C and pH of 9.0 (Rosenbaum et al. 2021). Further studies showed that the lactate dehydrogenase-encoding gene, *ldh*, in *M. thermoacetica* was significantly up-regulated in the presence of lactate, indicating that its expression is induced by lactate (Rosenbaum et al. 2021).

Formate formation was also observed in *M. thermoacetica* (Fröstl et al. 1996; Holden 2009). It has been known that formate synthesis in microorganisms mainly depends on two pathways: the methyl branch of the WL pathway and the breakdown of pyruvate mediated by pyruvate formate lyase (PFL) (Pierce et al. 2008). *M. thermoacetica* possesses a complete WL pathway, and this pathway has been proven to play a role in formate production (Holden 2009). Furthermore, multiple PFL-encoding genes have been identified in *M. thermoacetica* (Islam et al. 2015), indicating that the PFL-catalyzed pyruvate breakdown may also contribute to formate formation in this bacterium.

The synthesis of non-native products has been achieved in *M. thermoacetica*. Through the introduction of an acetone synthetic pathway, this bacterium could produce acetone directly from CO_2_ (Kato et al. 2021). Interestingly, the supplementation of additional electron acceptors could enhance cell growth and acetone production of *M. thermoacetica* in gas fermentation, in which dimethyl sulfoxide (DMSO) exhibited the most significant promotion effect, leading to an acetone yield of 4 mM (Takemura et al. 2023).

## Energy metabolism and oxidative stress response of *M. thermoacetica*

Hydrogen is the simplest electron donor in biological systems. Hydrogenases catalyze the reversible oxidation of H_2_, allowing bacteria to use H_2_ as an energy source to support their growth. *M. thermoacetica* has two classes of hydrogenases, namely, [NiFe]-hydrogenases and [FeFe]-hydrogenases (HydABC) (Huang et al. 2012; Pierce et al. 2008; Wang et al. 2013). However, these hydrogenases exhibited completely different activities under various cultivation conditions (Drake 1982; Huang et al. 2012; Kellum and Drake 1984; Wang et al. 2013). An electron-bifurcating [FeFe]-hydrogenase identified in *M. thermoacetica* was found to efficiently catalyze both the formation and uptake of H_2_ (Wang et al. 2013). When *M. thermoacetica* is grown on H_2_ and CO_2_, hydrogenases are employed to catalyze the H_2_ uptake, providing energy source to support its autotrophic growth (Drake and Daniel 2004; Kellum and Drake 1984; Martin et al. 1983); however, in the fermentation of sugars, such as glucose, these hydrogenases preferentially catalyzed H_2_ production, utilizing the energy released from glycolysis (Huang et al. 2012). Obviously, such a reversible hydrogenase activity enables *M. thermoacetica* to flexibly balance the intracellular redox state to adapt to different external environment.

Two potential energy conservation systems based on hydrogenase have been proposed in *M. thermoacetica* (Mock et al. 2014; Schuchmann and Müller 2014): (i) The energy-converting hydrogenase (ECH) complex, which consists of electron carrier proteins and [NiFe]-hydrogenases. The ECH-encoding genes are normally adjacent to formate dehydrogenase-encoding genes in the chromosome of acetogens (Mock et al. 2014). ECH and formate dehydrogenases can form a formate hydrogen lyase complex, which, coupled with methylene tetrahydrofuran reductase, enables the transfer of protons outside cell membrane, thereby creating a proton gradient across the cell membrane for ATP generation (Huang et al. 2012; Mock et al. 2014; Schuchmann and Müller 2014). (ii) A cascade reaction mediated by the ECH complex, ferredoxin hydrogenase, and methylene tetrahydrofuran reductase, which transfers intracellular protons to external environments, forming a proton gradient across cell membrane for ATP generation (Schuchmann and Müller 2014). However, functional identification and characterization of these energy conservation systems in *M. thermoacetica* have not yet been reported.

In addition, multiple genes responsible for the synthesis of cytochrome and menaquinone have been reported in *M. thermoacetica* (Bengelsdorf et al. 2015; Islam et al. 2015; Pierce et al. 2008; Poehlein et al. 2015; Redl et al. 2020; Tsukahara et al. 2014). Cytochrome and menaquinone can assist in transferring electrons to methylenetetrahydrofolate, a key intermediate product of the methyl branch of WL pathway in acetogens, thereby driving the operation of this carbon fixation pathway (Müller 2003; Schuchmann and Müller 2014). Such a cytochrome or menaquinone-mediated electron transport chain is likely to work in *M. thermoacetica* despite the lack of experimental evidences. Furthermore, electron bifurcation was also proposed to be involved in the energy metabolism of *M. thermoacetica* (Huang et al. 2012). The electron-bifurcating enzyme, NfnAB, was characterized and suggested to electronically connect the oxidative hexose metabolism (e.g., glucose) and reductive CO_2_ fixation (Huang et al. 2012). This process is crucial for the recycling of CO_2_ that is generated during glycolysis in *M. thermoacetica*, leading to a higher carbon yield compared to typical heterotrophic bacteria.

The supply and balance of reducing power are crucial for bacterial growth and product synthesis on CO_2_/CO. Excessive intracellular NADH level was found to inhibit the growth of *M. thermoacetica* (Kobayashi et al. 2022). Thus, the reversible hydrogenase activity, as mentioned above, is crucial for *M. thermoacetica* to balance the intracellular redox state. In addition, *M. thermoacetica* was reported to employ some fermentative products, such as glycerol as electron sink to dispose of excess reducing power (Kimura et al. 2016), and further use dimethyl sulfoxide (DMSO) and nitrite as the energy-conserving electron acceptors (Rosenbaum et al. 2022; Seifritz et al. 2003); these findings, although the underlying mechanisms in biochemistry and bioenergetics remains poorly explored, provide references to the optimization of metabolic flux in *M. thermoacetica*. Noticeably, microbial electrosynthesis (MES) has also been applied to *M. thermoacetica* for CO_2_ utilization, enabling the electroactive *M. thermoacetica* strains to employ extracellular electron mediators to reduce CO_2_ and generated acetate (Ha et al. 2022). This alternative approach expands the potential applications of *M. thermoacetica* in CO_2_ utilization.

Oxygen can significantly change the intracellular redox potential of anaerobic bacteria, thereby modifying the structures of some anaerobic enzymes and destroying their activities (Buckel 2021). Some enzymes such as methylene–THF reductase (MetfR), carbon monoxide dehydrogenase (CODH), and hydrogenase in the WL pathway are sensitive to oxygen (Ragsdale 1991; Whitham et al. 2015). *M. thermoacetica* has at least three approaches to survive in response to oxidative stress (Drake and Daniel 2004), i.e., the expression of enzymes catalyzing O_2_ scavenging, switching off the O_2_-sensitive acetyl-CoA pathway via nitrate dissimilation, and formation of a symbiotic relationship with facultative or facultative bacteria that can consume O_2_.

## Genetic tools and manipulations of *M. thermoacetica*

The exploitation of real potential of *M. thermoacetica* in the utilization of C1 gases relies on strain improvement via genetic manipulation. The first step towards this goal depends on the introduction of exogenous DNA into host cells, which requires overcoming the restriction modification barrier in the target bacteria. In 2013, exogenous plasmids, which were methylated in advance in *E. coli* by expressing multiple methyltransferase-encoding genes from *M. thermoacetica*, were successfully transferred into this bacterium via electroporation (Kita et al. 2013). Furthermore, a high-throughput method capable of coupling methyltransferases with their respective motifs was reported, through which a total of 11 sets of methylation modification systems (two for type I, seven for type II, and two for type III modification systems) were verified in *M. thermoacetica* ATCC 39073 (Bourgade et al. 2022; Jensen et al. 2019). This tool enabled systematic identification of methyltransferase recognition and modification patterns in *M. thermoacetica*, offering valuable clues for the further optimization of exogenous DNA transformation in this bacterium.

Due to the lack of suitable replicons, the early genetic manipulations in *M. thermoacetica* were based on non-replicating plasmid vectors. Recently, a replicating shuttle vector was constructed for *M. thermoacetica* using a compatible replicon from the *Thermotoga petrophila* pRKU1 plasmid (Bourgade et al. 2022). Based on this shuttle vector, the ethanol synthesis pathway in *M. thermoacetica* was strengthened by overexpressing an aldehyde dehydrogenase-encoding gene (*aldh*) and a bi-functional aldehyde–alcohol dehydrogenase-encoding gene (*adhE1*), leading to enhanced ethanol production (Bourgade et al. 2022). In addition, it is difficult to use antibiotic resistance as a selective marker for gene transfer in *M. thermoacetica,* because most antibiotic resistance enzymes are not stable under the high growth temperature (45–65 °C) of this thermophilic bacterium (Basen and Muller 2017). To overcome this limitation, a thermostable kanamycin resistant gene from *Streptococcus faecalis* was identified and used in *M. thermoacetica*, giving the kanamycin resistance to transformed cells (Iwasaki et al. 2013). Furthermore, an uracil auxotroph strain of *M. thermoacetica* was also generated as a host for genetic manipulations using uracil as the selection agent (Kita et al. 2013).

In summary, there have been limited examples demonstrating gene deletion, overexpression, and chromosomal integration of DNA fragments in *M. thermoacetica* (Daniel and Drake 1993; Iwasaki et al. 2017; Kato et al. 2021; Kita et al. 2013). Obviously, the genetic tools currently available for *M. thermoacetica* are far inferior to those of well-studied model bacteria. For example, the gene deletion in this bacterium still relies on the traditional homologous recombination rather than the more efficient CRISPR–Cas systems. Thus, it is urgent to expand and optimize the toolbox of *M. thermoacetica* for more efficient strain improvement.

## Optimization of gas fermentation of *M. thermoacetica*

Gas composition is crucial for the autotrophic growth and product synthesis of *M. thermoacetica*. The maximum biomass of *M. thermoacetica* could reach OD_600_ of ~ 11.3 when it was grown on a mixture of CO and CO_2_ (CO/CO_2_ = 7/3, 1000 sccm, pH6.0), leading to the formation of 31 g/L of acetate and a production rate of 0.55 g/L/h (Hu et al. 2013). When the gas was changed to a mixture of H_2_ and CO_2_ (H_2_/CO_2_ = 7/3), substantial amounts of acetic acid could still be produced (~ 30 g/L), but the maximum cell density (OD_600_) was only ~ 2.5 (Hu et al. 2016). The components of medium (e.g., vitamins, trace elements, and mineral elements) as well as cultivation conditions were also crucial for the performance of *M. thermoacetica* in gas fermentation (Hu et al. 2013). For instance, the supplementation of electron sink compounds such as glycerol and dimethyl sulfoxide (DMSO) could enhance cell growth and product formation in *M. thermoacetica* (Kimura et al. 2016; Rosenbaum et al. 2022).

The repeated-batch culturing with cell recycling has been adopted to enhance the biomass of *M. thermoacetica* in gas fermentation. Through this approach, the inhibition of acetate to *M. thermoacetica* was relieved, leading to the production of 840 mM acetate in gas fermentation (H_2_/CO_2_ = 4/1, pH6.2) (Sakai et al. 2005). Immobilization of cells offers advantages over free cells to gas fermentation, particularly in cell density and productivity. The *M. thermoacetica* cells have been successfully immobilized by calcium alginate embedding for semi-continuous gas fermentation in a bubble column bioreactor, leading to efficient production of acetic acid with a concentration of 32.3 g/L and a productivity of 2.13 g/L/d (Zhang et al. 2021). Noticeably,, to efficiently convert syngas (CO/CO_2_ or H_2_/CO_2_) into lipids that can be further used for biodiesel production, an integrated conversion approach comprising two stages was presented, in which *M. thermoacetica* was employed to convert syngas to acetic acid followed with the conversion of acetic acid into lipids by an engineered oleaginous yeast (*Yarrowia lipolytica*) (Hu et al. 2016). Such an integrated continuous reactor system could produce 18 g/L of C16–C18 triacylglycerides from syngas with an overall productivity of 0.19 g L^−1^ h^−1^ and a lipid content of 36% (Hu et al. 2016). Furthermore, an in situ nuclear magnetic resonance (NMR) bioreactor has been developed to monitor the metabolism of *M. thermoacetica* in real time, enabling the metabolic profiling of this bacterium throughout the whole fermentation process (Xue et al. 2014). This reactor system will contribute to the optimization of *M. thermoacetica*’s biosynthesis based on C1 gases.

Specific to industrial off-gases, they often contain different impurities, such as tar, ammonia, sulfide, and nitrogen oxide, which are generated during gas preparation process (Xu et al. 2011). These impurities tend to accumulate in the media during gas fermentation, thus affecting cell growth and product synthesis (Oliveira et al. 2022; Xu et al. 2011). For example, the ammonia in syngas can be rapidly converted into ammonium ion (NH_4_^+^), an inhibitor for hydrogenase activity, in fermentation and then impair the cell growth of *Clostridium ragsdalei* (Xu and Lewis 2012). Nitrogen oxide (e.g., NO) is also an inhibitor to hydrogenase, and just a small amount (above 40 ppm) of NO in syngas could repress the growth of acetogens (Ahmed and Lewis 2007). Therefore, both gas pretreatment (removing impurities) and enhancing the robustness of *M. thermoacetica* are crucial for its utilization of industrial off-gases.

## Conclusions and future prospects

Microbial fixation and conversion of C1 gases are poised to play an important role in green biomanufacturing. As a representative autotrophic acetogen, *M. thermoacetica* has shown immense potential for industrial production of commodity chemicals using CO_2_/CO. However, due to the poor understanding and insufficient genetic tools, the progresses in improving its metabolic capabilities have been limited to date. It is imperative to establish more efficient genetic tools for *M. thermoacetica*, particularly CRISPR-Cas-based genome editing and the derivative high-throughput screening technologies, such as pooled CRISPRi screening (Wang et al. 2018). This will help to accelerate our understanding of *M. thermoacetica* and strain modification for desired phenotypic traits.

From the perspective of industrial applications, construction of artificial cell factories of *M. thermoacetica* for direct conversion of C1 gases into value-added products is the preferred route. Simultaneously, other approaches are also worth considering. For example, given the superior ability of *M. thermoacetica* in acetate synthesis, an attractive route is functional cooperation of the acetate production of *M. thermoacetica* grown on C1 gases and the acetate conversion mediated by other microorganisms, such as *E. coli* or yeasts. Such an integrated process will bypass the obstacles in genetic modification of *M. thermoacetica*, thereby enabling efficient synthesis of various value-added chemicals from C1 gases.

## Data Availability

All data and materials are included in the article.

## Abbreviations

WL: Wood–Ljungdahl
C1: One-carbon
CO_2_: Carbon dioxide
CO: Carbon monoxide
MetfR: Methylene–THF reductase
CODH: Carbon monoxide dehydrogenase
NfnAB: NADH-dependent reduced ferredoxin: NADP oxidoreductase
ECH: Energy-converting hydrogenase
DMSO: Dimethyl sulfoxide
PFL: Pyruvate formate lyase
